# The clinical features and outcomes of systemic light chain amyloidosis with hepatic involvement

**DOI:** 10.1080/07853890.2022.2069281

**Published:** 2022-04-28

**Authors:** Liang Zhao, Guisheng Ren, Jinzhou Guo, Wencui Chen, Weiwei Xu, Xianghua Huang

**Affiliations:** National Clinical Research Center of Kidney Disease, Jinling Hospital, Nanjing University School of Medicine, Nanjing, China

**Keywords:** Hepatic amyloidosis, light chain amyloidosis, clinical manifestation, prognosis

## Abstract

**Objectives:**

To evaluate the clinical characteristics and prognostic factors of hepatic systemic light chain (AL) amyloidosis.

**Methods:**

Eighty-eight patients diagnosed AL amyloidosis with hepatic involvement between June 2004 and January 2019 were analysed retrospectively.

**Results:**

The median age of the patients was 55 years old, and the male to female ratio was 2.8:1.The main clinical manifestations include edema, digestive symptoms, weight loss, fatigue and ascites. Fifty-one patients received treatment, 42 patients were suitable for therapeutic efficacy evaluation and 25 (59.5%) achieved haematologic response. The median survival time was nine months, and the survival rates at one year, three years and five years were 33.0%, 11.4% and 6.8%, respectively. The risk of death was 6.6 times that of those who did not achieve haematologic response. Multivariate analysis showed that baseline NT-proBNP ≥ 1800 pg/ml and total bilirubin ≥ 34.2 umol/L were predictive of all-cause death.

**Conclusions:**

Systemic light chain amyloidosis with hepatic involvement is associated with poor survival but rarely has specific manifestations. The significant increase of NT-proBNP and hyperbilirubinemia indicate a poor prognosis. Vigilance should be raised to the relevant clinical manifestations, early diagnosis and timely treatment can improve the prognosis.

**KEY MESSAGES**
- Systemic light chain amyloidosis with hepatic involvement is associated with poor survival but rarely has specific manifestations.
- The significant increase of NT-proBNP and hyperbilirubinemia indicate a poor prognosis.

## Introduction

AL amyloidosis is a multisystem disorder characterized by the extracellular deposition of insoluble beta-pleated protein fibrils derived from misfolded monoclonal immunoglobulin light chain [1]. The liver is a major visceral organ of amyloid deposition, and histological evidence of liver involvement have been observed in about 70% of the cases with AL amyloidosis in one autopsy series [2], while clinically significant hepatic involvement is uncommon, with only around 20–30% of patients have clinical evidence of liver involvement [3,4]. The manifestations of hepatic involvement are varied, including fatigue, weight loss, decreased appetite, abdominal pain, ascites, hepatomegaly and elevated alkaline phosphatase (AKP) level. In rare cases, hepatic amyloidosis may also cause jaundice, spontaneous rupture of the liver and liver failure, result in fatal consequence [5–7]. The confirmation of hepatic involvement is often delayed due to the fact that the presenting symptoms are often mild or mimic other more common conditions.

The clinical outcome of hepatic AL amyloidosis is poor, with a median survival time of only 8.5 months in patients with biopsy-proven hepatic involvement [8]. And the 5-year and 10-year survival rates are reported as 13.0–16.9% and 1.0–6.6%, respectively [8,9]. The prognosis is even worse in patients with hyperbilirubinemia and jaundice [7]. Fortunately, in the current era, the survival of AL amyloidosis patients has improved with advances in diagnosis, treatment options and response assessment methods [10]. Similarly, in hepatic AL amyloidosis patients, liver transplant (LT) and high-dose intravenous melphalan and autologous stem cell transplantation (HDM/ASCT) has been shown to be associated with positive outcomes [11–13]. Despite the advance in the disease management, few attempts have been made to analyse the prognosis of AL patients with hepatic involvement, especially after the introduction of HDM/ASCT and novel drugs, as well as new prognostic biomarkers and risk stratification systems in the new century [14,15]. For this reason, we have carried out a retrospective survey of clinical features, prognostic factors and survival of AL amyloidosis patients with liver involvement.

## Methods

### Study population

Retrospectively we reviewed 88 cases of patients with AL amyloidosis and predominant hepatic involvement who were newly diagnosed in our institution between June 2004 and January 2019. The diagnosis and type of AL was histologically confirmed (Congo-red, immunofluorescence). The histologic specimens were obtained by using at least one of the following biopsies: abdominal skin and fat, rectum mucosa, bone marrow, renal or liver. Plasma cell dyscrasia was documented by serum immunofixation electrophoresis and serum-free light chain test. Patients with ATTR, AA, familial or localized (including dialysis-related) amyloidosis were excluded. This study followed the Declaration of Helsinki Ethical Principles for Medical Research involving human subjects. Informed consent was obtained from each participant to have their medical records reviewed, and the study was approved by the institutional ethics review board of Jinling Hospital.

Information on demographic, clinical and laboratory data were collected from electronic medical records of patients. The assessment of organ involvement was based on the 2005 International Society of Amyloidosis guidelines [16]. Specifically, liver involvement was defined as total liver span > 15 cm in the absence of heart failure or AKP > 1.5 times institutional upper limit of normal or liver biopsy-proven amyloidosis. To calculate the number of organs involved, only heart, kidney, liver and nerve were included. The selection criteria for ASCT were shown in Table 1. The ASCT protocol included mobilization with colony-stimulating actor alone and conditioning with high-dose melphalan 140–200 mg/m^2^. Both the 2004 and 2012 Mayo AL amyloidosis staging systems were calculated for all patients. Haematologic and organ responses were assessed according to the consensus criteria three and/or six months after treatment initiation [17]. In particular, hepatic response was defined as greater than or equal to 50% decrease of an AKP level and/or greater than or equal to 2 cm decrease in liver size (assessed by radiography). The eGFR was calculated using the Chronic Kidney Disease Epidemiology Collaboration (CKD-EPI) equation. Performance status was graded according to the Eastern Cooperative Oncology Group.

**Table 1.** Selection criteria for autologous stem cell transplantation.

	Inclusion criteria	Exclusion criteria
Age (years)	18–70	< 18 or >70
ECOG-PS	0–2	> 2
LVEF (%)	> 45	≤45
TBIL (mg/dl)	≤2	> 2
Creatinine(mg/dl)	≤2	> 2
Active infection	No	Yes

ECOG-PS: Eastern Cooperative Oncology Group performance status; LVEF: left ventricular ejection fraction; TBIL: total bilirubin.

### Statistical analysis

Continuous variables were checked for normality using the Kolmogorov–Smirnov test and described with mean ± SD or with medians and interquartile ranges as appropriate. Categorical variables were presented as absolute numbers and percentages. Variables were dichotomized according to the prognostic cut-offs previously reported for hepatic amyloidosis or all the AL amyloidosis [8,14,15] or dichotomized by their median values. The endpoint for prognostic analysis was overall survival (OS), which was calculated from the date of diagnosis to the date of all-cause death. Data were censored for subjects who were still alive at last follow-up or at the cut-off date (Sep 15, 2019). Survival curves were plotted with Kaplan–Meier method and compared with the log-rank test. Cox proportional hazards models were constructed to calculate hazard ratios (HRs) and 95% confidence intervals (CIs), and variables with *p* values < .1 in univariate analysis were considered for multivariate analyses. Statistical significance was set at *p* < .05. Analyses were performed using the SPSS 20 software (IBM SPSS statistics for Windows, Version 20.0, IBM Corp, Armonk, NY, USA). GraphPad prism v.8.0.0 (GraphPad Software Inc, San Diego, CA) was used to generate figures.

## Results

### Baseline characteristics

The median age of 88 patients was 55 years, and 73.9% of them were males. The most common clinical manifestations were edema, and 78 (88.6%) patients showed different degrees of edema; digestive symptoms were observed in 59 (67.0%) patients, including anorexia, abdominal distention, diarrhoea, nausea and vomiting; weight loss appeared in 51 (58.0%) patients with an average weight loss of 7.2 kg at diagnosis; fatigue was manifested in 49 (55.7%) patients; congestive heart failure was found in 12 patients (13.6%). Liver enlargement was found in 49 (55.7%) patients and splenomegaly in 12 (13.6%) patients. The median liver span was 135 mm (IQR 119, 157). The median AKP was 415 U/L (IQR 308, 574). The median serum bilirubin level was 9.7 umol/L (IQR 6.3, 15.4). Forty-six patients (56.8%) presented with ascites. Spontaneous splenic rupture occurred in one patient, he underwent splenectomy and HDM/ASCT successively and survived until the follow-up deadline.

There were 69 patients with λ and 19 patients with κ clonal plasma cell dyscrasia including 4 cases with clinically overt multiple myeloma; and the ratio of λ type to κ type was 3.6:1. This distribution of the ratio of λ:κ light chain is similar to that of the entire group of AL amyloidosis patients (λ: κ = 3.8) [18]. Due to our institution is a kidney disease centre, there were 87 patients having kidney involvement in this study. After liver and kidney, the most commonly involved visceral organ was the heart (75.0%). Mayo AL amyloidosis 2012 stage I/II/III/IV (18/28/31/11), Mayo AL amyloidosis 2004 stage I/II/III (14/23/51). Six patients had serological evidence of chronic hepatitis B infection, while virus DNA replication was found in none. No patient had serological evidence of chronic hepatitis C infection. The other major baseline characteristics of 88 patients were displayed in Table 2.

**Table 2.** Baseline characteristics.

	Characteristics (*n* = 81)
Age at presentation (years)	55 (47, 63)
Male (%)	65 (73.9)
ECOG-PS (%)	
0	0 (0.0)
1	33 (37.5)
2	36 (40.9)
3	15 (17.0)
4	4 (4.5)
Organ involvement (%)	
Renal	87 (98.9)
Liver	88 (100)
Cardiac	66 (75.0)
Hypotension	28 (31.8)
MAP (mmHg)	85 ± 14
AKP (U/L)	415 (308, 574)
AK*p* ≥ 500 U/L	30 (34.1)
TBIL (umol/L)	9.7 (6.3, 15.4)
TBI*L* ≥ 34.2 umol/L	9 (10.2)
AST (U/L)	48 (36, 71)
ALT (U/L)	40 (25, 57)
LDH (U/L)	266(218, 357)
Total liver span (mm)	135 (119, 159)
TC (mmol/L)	8.6 ± 5.1
Platelets (×10^9^/L)	305 ± 132
Platelet*s* ≥ 500 × 10^9^/L)	8 (9.1)
PT (s)	12.3 ± 1.8
cTnT (ng/ml)	0.048 (0.028–0.139)
cTn*T* ≥ 0.025 ng/ml	54
cTn*T* ≥ 0.035 ng/ml	30
NT-proBNP (pg/ml)	1630 (536–4127)
NT-proBN*p* ≥ 1800 pg/ml	31 (35.2)
Mayo 2004 stage I/II/III	14/23/51
Mayo 2012 stage I/II/III/IV	18/28/31/11
IVST (mm)	12 ± 3
LVEF (%)	64 ± 8
dFLC (mg/L) (*n* = 80)	94.0 (33.4–139.3)
dFL*C* ≥ 180 mg/L	15
BMPC (%)	2.5 (0.5–5)
Uric acid (umol/L)	423 ± 145
Albumin (g/L)	26.0 ± 6.3
Creatinine (mg/dl)	1.10 (0.84, 1.90)
24 h urine protein (g)	4.40 (2.50, 7.51)

Values are given as mean ± SEM, median (interquartile range) or percentage. ECOG-PS: Eastern Cooperative Oncology Group performance status; MAP: mean arterial pressure; AKP: alkaline phosphatase; TBIL: total bilirubin; AST: aspartic aminotransferase; ALT: alaninetransaminase; LDH: lactate dehydrogenase; TC: total cholesterol; PT: prothrombin time; cTnT: cardiac troponin T; NT-proBNP: N-terminal natriuretic peptide type B; IVST: interventricular septum thickness; LVEF: left ventricular ejection fraction; dFLC: difference between involved and uninvolved free light chains; BMPC: bone marrow plasma cells.

### Treatment and response

Fifty-one (58.0%) patients received treatment; 15 patients received high-dose melphalan and HDM/ASCT, The median of N-terminal pro-brain natriuretic peptide (NT-proBNP) in patients receiving ASCT was 541 pg/ml (range 33.88–1724 pg/ml). Eight patients received a melphalan dose of 200 mg/m^2^ and seven patients received a dose of 140 mg/m^2^. Twenty-six patients received bortezomib-based chemotherapy, and 19 patients received other chemotherapy regimens (including melphalan- and thalidomide-based chemotherapy). Forty-two patients were suitable for therapeutic efficacy evaluation (treatment lasted for at least three months). Twenty-five (59.5%) patients achieved haematologic response, of which 17 (40.5%) achieved haematologic complete response (CR), with a median remission time of one month, 7 (16.7%) achieved very good partial response (VGPR), with a median remission time of one month, and 1 (2.9%) patient achieved partial response (PR) at one month. Of 15 patients received HDM/ASCT treatment, hyperbilirubinemia was found in only 1 patient and his baseline total bilirubin level was 27.1 umol/L. His serum total bilirubin level dropped to 9.6 umol/L after ASCT. One patient died of splenic rupture during stem cell transplantation. Eleven patients received induction treatment before ASCT, eight with bortezomib-based regiment, two with thalidomide-based regiment and one with VAD (vincristine Adriamycin Dexamethasone) regiment, and eight of them achieved haematologic response (five with CR and three with VGPR). Among 15 patients received ASCT, 14 (93.3%) patients achieved haematologic remission with 10 (66.7%) patients achieved CR, eventually. With regard to organ response, 15 (37.5%) patients achieved renal response and 15 (35.7%) patients achieved liver response with a median remission time of 6 months and 10 months, respectively. Among the 42 patients, 31 patients had heart involvement, and 11 (35.5%) patients achieved cardiac response with a median remission time of five months. The haematologic and organ responses after each treatment was shown in Table 3.

**Table 3.** Haematologic and organ responses.

	HDM/ASCT	Bortezomib-based regimen	Other chemotherapy regimen^a^
	*n* = 15	*n* = 24	*n* = 9
Haematologic response ≥ PR (%, n)	92.9(14/15)	62.5(15/24)	22.2(2/9)
Median haematologic response time (month)	1	2	1
Liver response (%, n)	60.0(9/15)	37.5(9/24)	11.1(1/9)
Median liver response time (month)	7.5	16	3
Kidney response (%, n)	73.3(11/15)	50.0(12/24)	11.1(1/9)
Median kidney response time (month)	6	6	7
Heart response (%, n)	77.8(7/9)	33.3(8/24)	14.3(1/7)
Median heart response time (month)	8	3	8

HDM/ASCT: high-dose intravenous melphalan and autologous stem cell transplantation; PR: partial response.

^a^Other chemotherapy agents include melphalan, thalidomide-based chemotherapy.

### Patient survival

**Figure 1.**
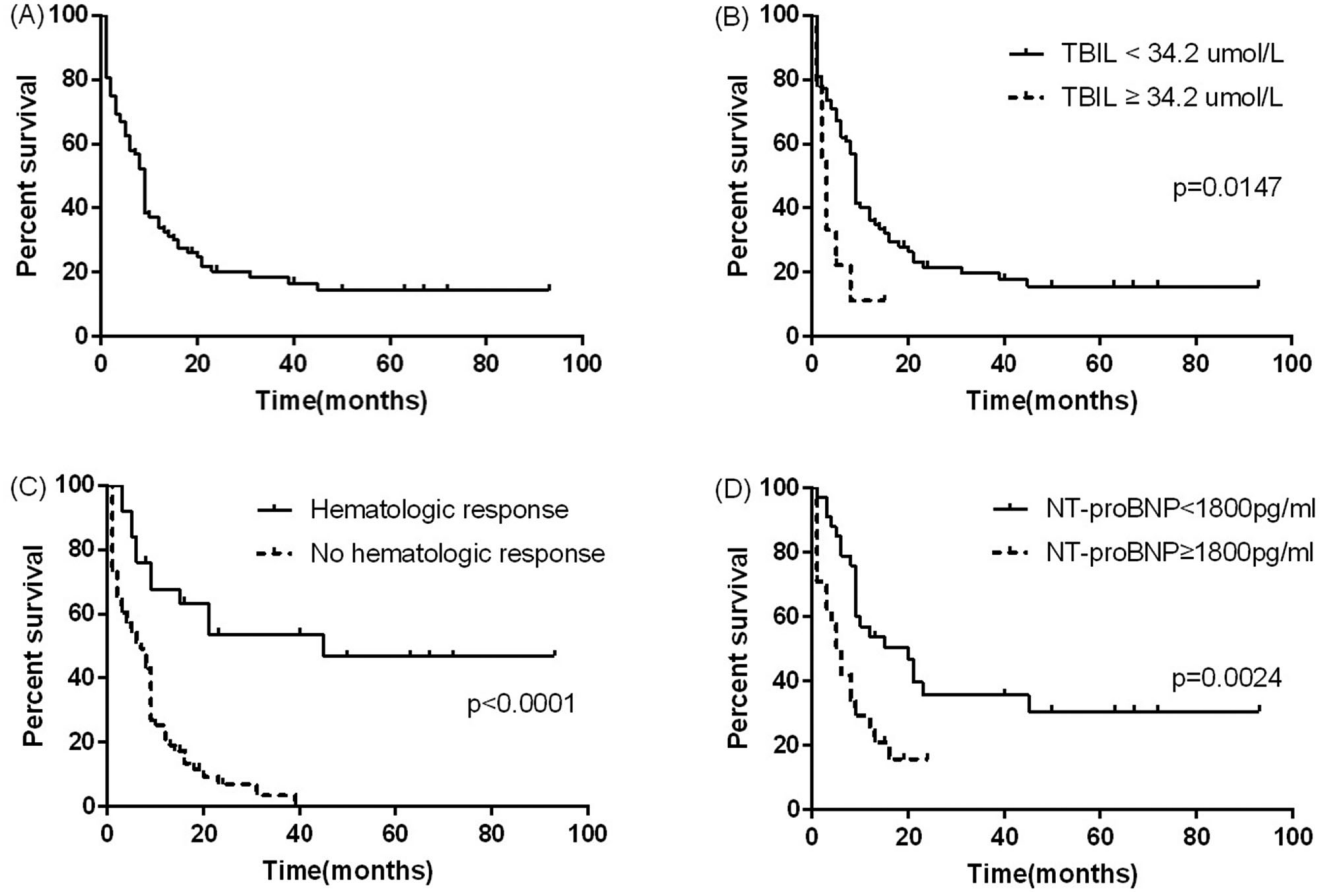
(A) The OS of all patients. (B) The OS between patients who had total TBil ≥ 34.2 umol/L and TBil < 34.2 umol/L. (C) The OS between patients who had haematologic response and no haematologic response patients. (D) The OS between patients who had NT-ProBNP ≥ 1800 pg/ml and NT-ProBNP <1800 pg/ml.

During the median follow-up period of nine months, 72 (81.8%) patients died. The median OS after diagnosis was nine months (95% CI: 7.896–10.104). The survival rates at one year, three years and five years were 33.0%, 11.4% and 6.8% (Figure 1). The mortality risk of patients without haematologic remission was 6.6 times that of patients with haematologic remission (95% CI: 2.8–24.1, *p* = .001). The median survival time of patients without haematologic remission was 8 months (95% CI: 6.2–9.8), and the median survival time of patients with haematologic remission was 45 months (95% CI: 21.7–88.3) (*p* < .001) (Figure 1). The one-year, three-year and five-year survival rates of patients with haematologic remission were 68.0%, 56.0% and 52.0% respectively.

There were eight variables to be consider in univariate analysis. In univariate analysis, age ≥ 55 years old, total bilirubin ≥ 34.2 umol/L, NT-proBNP≥ 1800 pg/ml yielded a *p* value below .1 and so were included in the multivariate analysis. In multivariate analysis, baseline NT-proBNP ≥ 1800 pg/ml (HR 2.30, 95% CI: 1.19–4.44, *p* = .013) and total bilirubin ≥ 34.2 umol/L (HR 3.29, 95% CI: 1.07–10.07, *p* = .0373) were predictive of all-cause death (Table 4). Survival curve of the patients with NT-proBNP ≥ 1800 pg/ml was significantly different from that of patients with NT-proBNP less than 1800 pg/ml (*p* = .004) (Figure 1). Kaplan–Meier curves for patients with total bilirubin ≥ 34.2 umol/L and total bilirubin < 34.2 umol/L also showed similar results (*p* < .0001) (Figure 1).

**Table 4.** Results of univariate and multivariate analysis for all-cause mortality.

Variable	HR (95% CI)	*p* value
Univariate		
Age ≥ 55岁	1.59 (0.99–2.53)	.053
ECOG-PS	1.23 (0.91–1.64)	.175
Hypotension	1.37 (0.84–2.23)	.200
TBIL ≥ 34.2umol/L	3.38 (1.64–6.99)	.001
AKp ≥ 500 U/L	1.48 (0.91–2.41)	.112
NT-proBNp ≥ 1800 pg/ml	2.40 (1.27–4.52)	.007
c-TnT ≥ 0.025 ng/ml	1.70 (0.76–3.78)	.196
dFLC ≥ 94 mg/L	0.78 (0.49–1.25)	.297
Multivariate		
TBIL ≥ 34.2umol/L	3.29 (1.07–10.07)	.037
NT-proBNp ≥ 1800 pg/ml	2.30 (1.19–4.44)	.013

HR: hazard ratio; CI: confidence interval; ECOG-PS: Eastern Cooperative Oncology Group performance status; TBIL: total bilirubin; AKP: alkaline phosphatase; NT-proBNP: N-terminal natriuretic peptide type B; cTnT: cardiac troponin T; dFLC: difference between involved and uninvolved free light chains.

## Discussion

Even though hepatic involvement is very common in AL amyloidosis and is associated with poor prognoses [8,9], it has not received as much attention as heart involvement. A majority of studies on the liver involvement were case reports. Few large-sample studies have been focussed on the prognostic risk factor of hepatic involvement. Park et al. published the largest cohort of AL amyloidosis patients with hepatic involvement and found that congestive heart failure before biopsy, total bilirubin > 34 umol/L and platelet count > 500 × 10^9^/L were predictors of a poor prognosis. However, patients included in the study were diagnosed before 1997, making it impossible to evaluate the prognostic impact of HDM/ASCT and bortezomib-based chemotherapy, as well as some new biomarkers such as NT-proBNP, cTnT and free light chain (FLC) [8]. Therefore, we thoroughly investigated the clinical and prognostic characteristics of liver AL amyloidosis in this study. The results showed that the prognosis of patients with liver involvement is still poor in the new era of AL amyloidosis management, with a median OS of 9 months, which was consistent with those reported previously [8,9]. Baseline NT-proBNP ≥ 1800 pg/ml and total bilirubin ≥ 34.2 umol/L were independent risk factors for mortality and achieving haematologic remission was a strong protective factor regardless of therapeutic regimen.

With regard to the clinical manifestations of our cases, the most common symptoms were edoema, involuntary weight loss and fatigue. Other less common symptoms were anorexia, abdominal distention, nausea and vomiting and congestive heart failure. These non-specific symptoms were similar with other studies [8,11]. However, the average weight loss was lower than reported (7.2 kg vs. 10.4 kg) [8]. This might be due to sodium and water retention and edoema caused by renal involvement. The most common physical findings were ascites and hepatomegaly, which were similar to those reported in other studies [8,13,19]. An elevation in serum AKP level is the most frequently abnormal blood test in hepatic AL amyloidosis. Park et al. reported 86% patients of AL amyloidosis with hepatic involvement had an increased AKP level and 61% had values of 500 U/L or more [8]. In our cases, however, AKP ≥ 500 UL/L was only found in 30 of 88 (34.1%) patients with a median value of 415 U/L. This might be partly due to the differences in the study population, because the upper normal reference range of AKP is lower in our laboratory than it was in the previous study (172 U/L vs 250 U/L). Gertz and Kyle reported a total bilirubin (TBIL) value of more than 1.5 mg/ml (25.7 umol/L) in 10 of 78 (12.8%) patients and exceeded 5 mg/dl in 3 of 78 (3.8%) patients, which were similar to our result [9]. But Park et al. reported that 21% of patients presented with TBIL > 34 umol/L [8]. Spontaneous rupture of the liver or spleen is rare but usually fatal complication [6]. Liver rupture was not observed in our cases, and spleen rupture was occurred in one case as previously described.

The median survival of patients in this study was nine months, which was similar to previous studies [8,9]. Hyperbilirubinemia and jaundice were suggested to confer a particularly poor prognosis in several articles [8,9,20,21]. Gertz and Kyle reported that the median survival time of patients with TBIL exceeding 1.5 mg/dL was only 1.8 months. Park et al. reported that TBIL > 34 umol/L, platelet > 500 × 10^9^/L and congestive heart failure were independent prognostic risk factors as mentioned before [8]. Our study confirms that TBIL ≥ 34.2 umol/L was indeed an independent risk factor affecting prognosis; however, only eight patients had baseline platelet count > 500 × 10^9^/L in our case, and platelet count was not associated with prognosis in univariate analysis. In addition, result showed that NT-ProBNP ≥ 1800 pg/ml was an independent risk factor for prognosis. NT-proBNP is a widely used biomarker for assessing the severity of heart failure and is used to establish risk stratification systems for AL amyloidosis and to judge the cardiac response after treatment [14,15,22]. Congestive heart failure was also proved to be an independent risk factor for prognosis of hepatic amyloidosis [8]. Therefore, it is reasonable to find that baseline NT-ProBNP ≥ 1800 pg/ml and TBIL ≥ 34.2 umol/L were useful for predicting the prognosis of patients with hepatic involvement.

In a study conducted on 69 patients with liver involvement who were treated with HDM/ASCT, Girnius et al. reported that 58 (84%) of patients achieved haematologic remission with 53% (31/58) haematologic CR at one year; hepatic response occurred in 57% (33/58) and 63% (19/30) of patients at one year and two years, respectively [12]. In our study, 25 of 42(59.59%) patients achieved haematologic remission. Of 15 patients receiving HDM/ASCT, 14 (93.3%) achieved haematologic remission, 10 (66.7%) obtained haematologic CR, the haematologic remission rate in this study was higher, possibly because some patients received bortezomib for induction therapy before HDM/ASCT as stated in our published article [23]. It is also important to note that patients undergoing HDM/ASCT must meet strict eligibility criteria [18]. Therefore, the excellent outcomes of these patients are somewhat confounded by selection bias. In the whole AL amyloidosis population received bortezomib-based regimen, a haematologic remission was achieved in 75% of patients and complete response was achieved in 45% of patients [24], which was higher than our result: 41.7% haematologic remission. This may be because some patients with hepatic involvement were too ill to tolerate the regimen or did not survive long enough for remission. Furthermore, our results showed that the efficacy of other chemotherapy regimens was poor for patients with hepatic involvement. In addition, we found that the death risk in patients without haematologic remission was 6.6 times higher than that in patients with haematologic remission, indicating that patients with haematologic remission would benefit for a long time regardless of the treatment regimen. Liver transplantation accompanied by chemotherapy or ASCT has been tried in patients with decompensated hepatic AL amyloidosis and may play a role in treatment of patients with advanced hepatic involvement in the future [11,13,19]. Yet, we have no experience with liver transplantation for the treatment of hepatic AL amyloidosis so far.

There are still some limitations in this study. First, this study is a single-centre retrospective study. Second, due to giving up treatment after confirmed diagnosis or the extremely short survival time of some patients, there are only 42 cases suitable for evaluating therapeutic efficacy. Therefore, the results of this study need to be further validated in a multi-centre prospective study.

In summary, hepatic involvement is associated with poor survival but rarely has specific manifestations. Baseline NT-ProBNP ≥ 1800 pg/ml and TBIL ≥ 34.2 umol/L were independent risk factors for AL amyloidosis with hepatic involvement in our cohort. Early diagnosis and appropriate treatment would be crucial to improve the prognosis.

## Data Availability

All data generated within this study are available from the corresponding author on request.
